# Cultivating diversity as an ethos with an anti-racism approach in the scientific enterprise

**DOI:** 10.1016/j.xhgg.2021.100052

**Published:** 2021-09-21

**Authors:** Shameka P. Thomas, Kiana Amini, K. Jameson Floyd, Rachele Willard, Faeben Wossenseged, Madison Keller, Jamil B. Scott, Khadijah E. Abdallah, Ashley Buscetta, Vence L. Bonham

**Affiliations:** 1 Social and Behavioral Research Branch, National Human Genome Research Institute, National Institutes of Health, Bethesda, MD 20892, USA; 2 Office of the Director, National Human Genome Research Institute, National Institutes of Health, Bethesda, MD 20892, USA

## Abstract

The diversity of the U.S. population is currently not reflected in the genomic workforce and across the greater scientific enterprise. Although diversity and inclusion efforts have focused on increasing the number of individuals from underrepresented groups across scientific fields, structural racism remains. Thus, the cultivation and adoption of diversity as an ethos requires shifting our focus to being intentional about an institution’s character, culture, and climate. One way for this ethos to be sustained is by facilitating an intentional anti-racism approach within the field. Adopting a new perspective on diversity utilizing an anti-racism approach will support genomics researchers as we build supportive, collaborative research environments. We seek to expand critical thought in the framing of diversity in the research enterprise and propose an anti-racism approach that informs deliberate actions required to address structural racism.

## Teaser

The cultivation and adoption of diversity in the workforce as an ethos requires shifting our focus to being intentional about an institution’s character, culture, and climate. One way for this ethos to be sustained is by facilitating an intentional anti-racism approach within genomic science.

## Introduction

Building diverse teams that reflect the U.S. population has been identified as an imperative in the field of genomics.^1^ In the U.S., the evidence is abundantly clear with regard to the social groups left out of the scientific workforce.^2^ As a diverse team of researchers, we present the merits of establishing diversity as an ethos in the field of genomics and the wider scientific community, utilizing an anti-racism approach. Teams with greater diversity among members can outperform homogeneous teams.^3^, ^4^, ^5^ Therefore, scientific researchers should be intentional about the process of team building in ways that are inclusive of the life experiences, expertise, backgrounds, and social identities within individual laboratories and across the field of genomics. Building a diverse team requires creating space for all team members to speak (and reflect) on how race and racism in the research enterprise affect and impact their lived experiences.

Structural racism continues to perpetuate inequities in representation across the scientific workforce, which limits scientific innovation.^6^, ^7^, ^8^ Addressing these gaps requires the consistent development of new and thoughtful team-building strategies, particularly in genetics, a scientific field with a troubling racist history.^9^, ^10^, ^11^ A stark example of this history is the “ideology of race,” a concept that has been foundational to the field of genetics, separating the human species into racial groups and reinforcing ideals of hierarchical superiority based upon “biological differences” among these groups. From this ideology the eugenics movement emerged, relying heavily on genetics to promote the philosophy of racial hierarchy.^9^^,^^12^ Today, genomic science continues to struggle with the misuse of race as discrete biological grouping.^13^ The worldwide reckoning with racism after the public murders of unarmed Black Americans, against the backdrop of the disproportionately deadly COVID-19 pandemic, has further revealed the structurally embedded injustices adversely impacting Black and Brown communities.^14^, ^15^, ^16^ The impact of xenophobia, violence, and racism in the United States is pervasive, including attacks on Asian American and additional marginalized communities.^17^^,^^18^ These social realities have made it tremendously clear that racism is not an archaic notion from years past. Rather, racism is deeply present across today’s social fabric, embedded into our structural institutions, and influences each of our lived experiences.^19^^,^^20^

In these critical times, we pause to ask the question: what does building a diverse team of researchers mean in the scientific workforce? In order for the field of genomics, and science at large, to be continuously innovative, we must be intentional in how we address structural barriers within the field that adversely affect team building. Comprehensive efforts to address the lack of workforce diversity are underway.^1^ As workforce diversity initiatives materialize and expand, individual researchers, labs, and departments in the genomics community are left with the question of how they can contribute to this call. We propose that adopting diversity as an ethos by utilizing an anti-racism approach should be used to answer this call.

## What does diversity as an ethos mean?

Ethos is the fundamental character, customs, ethics, and spirit of an institution that allows for its persons, ideas, and practices to exist or co-exist in synergy.^21^ Ethos represents not only the outward display of an institution’s character but also the inward display of an institution’s personality. Each institution or organization takes on an ethos or character that is continuously shaped by the social practices accepted (as norms) and/or ignored (as marginal).^21^, ^22^, ^23^, ^24^ This also considers how the institution’s past continues to inform its present-day policies and practices.^21^, ^22^, ^23^, ^24^

We apply this definition of ethos to expand our current thinking on diversity. Conventionally, diversity is framed in terms of representation,^25^ but we argue that it does not stop there. The key point is that diversity is not an outcome, but a process. It is not a one-time step, but a progression of steps that are intentionally built into the fabric of a lab, team, department, academic institution, or company over time.^24^ This course of action is consistently facilitated and encouraged by all members involved, not just by a select few. We identify three approaches the field should consider to shift toward diversity as an ethos.

### Valuing diversity beyond the numbers

First, while a lab or research team may look diverse in numbers, this does not necessarily suggest that the research environment is conducive for supporting scientists who identify as racial minorities.^26^ Selection of scientists at all stages (e.g., from trainees to principal investigators) is an essential effort to enhance diversity, but it cannot entirely redress decades of historical underrepresentation that has resulted from structural exclusion. Building a diverse team does not stop at increasing representation; rather, it starts there and should subsequently move toward efforts to consistently challenge the norms and conventions that perpetuate underrepresentation and exclusion. Diversity as an ethos therefore embodies a shift toward intentionality and introspection regarding an institution’s character, culture, and climate.

### Diversity is not a favor to underrepresented groups in science

Second, diversity as an ethos means that efforts to increase diversity should not be approached as a favor to underrepresented scientists. Talent and scientific creativity exist in all communities; therefore, researchers in leadership and hiring positions should hope for and intentionally establish opportunities to hire and train scientists from diverse backgrounds, because it would benefit the scientific enterprise.^2^^,^^27^ The tangible advantages of a diverse workforce are well-evidenced across a variety of disciplines.^3^^,^^28^, ^29^, ^30^ Freeman et al.^5^ found that papers co-authored by racially and ethnically diverse contributors led to greater contributions to science and across the canon (e.g., impact factors, citations, and the discourse) of knowledge production. This concretely demonstrates that increasing the scientific workforce diversity does not merely benefit individuals from underrepresented communities; greater workforce diversity advances scientific innovation and the scientific enterprise at large. Characterizing diversity as an indulgence or form of benevolence^31^ undermines the innovation and acumen that underrepresented scientists bring to scientific discovery. This kind of characterization can be found in diversity efforts centered around inclusion. While efforts framed around inclusion are important for achieving equity, this framing also often brandishes power dynamics: an expectation that those individuals “included” will assimilate into an existing hierarchical order.^32^ In contrast, diversity as an ethos centers the notion that the successful future of biomedical research relies on a diverse workforce, in which underrepresented scientists will also elevate and transform the field.^33^ Therefore, in order for the field of genomics to increase knowledge production and fulfill its innovative potential, the genomics workforce must become more diverse, as the future of the field depends on it.^1^

### Diversity requires challenging institutional norms

Third, diversity as an ethos is an ongoing and intentional process that calls for inward institutional introspection^24^ to challenge the norms that perpetuate underrepresentation, exclusion, and structural racism. This means not only hiring individuals from underrepresented backgrounds but also addressing the ways in which the larger institutional culture undermines and undervalues minoritized scientists within the field. To truly enhance the diversity of the workforce, we must reckon with the norms and practices^34^ that deepen the gaps in representation within the scientific enterprise. As Parikh^34^ comments, “diversity is a double-edge sword…. it is often the less obvious factors—divisive rhetoric, obsolete policies (such as overreliance on standardized tests), and willful blindness to inequitable treatment (such as smaller startup budgets for female academics)—that cement many of the injustices that have sprung from the nation's segregated history.”

Regardless of the intent of certain norms and practices, those that inherently symbolize and maintain exclusion and racism must be challenged to cultivate diversity as an ethos. Further examination of the larger institutional landscape of biomedical research reveals myriad examples of how certain norms and practices, such as those mentioned above, can lead to disparities in opportunity and perpetuate exclusion within the scientific field. Minority-group scholars, particularly Black scholars, are under-cited^35^ and less likely to receive R01 awards.^7^ The topics that African American and Black R01 applicants tend to propose to study—such as health disparities and research concerning socioeconomic and psychosocial factors—have been shown to receive less funding.^2^ Demographically underrepresented U.S. doctoral recipients generate significant novel science; however, the more underrepresented they are in their field, the less their novel contributions are adopted.^36^ Diversity as an ethos requires confronting the norms and practices that produce such inequitable outcomes. Moreover, it requires reflecting on how these norms and practices within the culture of science are being maintained and reproduced in one’s own individual research team. It considers how traditions and practices within an institution can perpetuate implicit bias, micro-aggressions, and racism in lab settings that can leave some scientists feeling undervalued, and even inclined to leave,^37^^,^^38^ contributing to issues of retention in the field.^39^

We contend that these key components—(1) valuing diversity beyond the numbers, (2) acknowledging that diversity is not a favor, and (3) reflecting on diversity by challenging institutional norms—are necessary for building an inclusive genomics and biomedical research community at large. Therefore, we argue that one way to facilitate this process of ethos development is to use an anti-racism approach.

## What does an anti-racism approach mean?

To define an anti-racism approach, we must first begin with: what is racism? Racism is an interwoven and hierarchical system used to organize structures and opportunities based on the values assigned, including nationality, ethnicity, phenotypic, or other markers of social difference and how these values are socially interpreted.^40^, ^41^, ^42^ These social interpretations construct a system that perpetuates implicit bias, discrimination, stereotypes, and stigmatization, which disproportionately disadvantages some groups and privileges other groups.^41^ Racism is a systemic and institutionally driven force that shapes and melds the foundations of social institutions and organizations and, in turn, shapes the socialization patterns of individuals.^42^ Structural racism is not solely about distinguishing between who is “racist” and who is not, but rather about how social patterns, social experiences, and social systems continue to divide groups of people across resources, accessibility, opportunities, and credibility.^43^ We identify three central tenants of an anti-racism approach.

### An anti-racism approach is intentional

Simply studying race or racism does not mean a team is approaching research through a lens of anti-racism. An anti-racism approach must be intentional. Naming anti-racism is a deliberate act of the researcher (or research team) and functions as a guiding mantra of the research team.^44^ Anti-racism, in this vein, is not about what type of research the lab is investigating, but it is about asking: (1) How are studies being conducted in this lab? (2) Who are the scientists conducting these studies? and (3) How does the research environment cultivate space for reflection across the research process, individually and collectively? Although these are just a few questions, we argue that this is an example of the type of intentionality to cultivate among researchers. This is important because everyone on a research team across all social identities is experiencing their own respective racial identity and perceived identity. An anti-racism approach thus asks, can members of the team talk about how their identities impact their research productivity, research processes, and experiences on the research team? More importantly, if (and when) racial minorities talk about their experiences when navigating the scientific enterprise—are they heard?

### An anti-racism approach is critically introspective

Anti-racism also requires humility^45^ and critical introspection. Introspection means that those who are often minoritized (and vulnerable) should not be the only individuals exercising humility. This means that humility and respect are extended to fellow colleagues through active listening and deep reflection. In other words, this is not just given to research study participants (during data collection) but also must be extended to teammates (on research projects).^46^ Critical introspection is less about answers and solutions and more about individuals (across the research enterprise) asking themselves questions as a method for strengthening their respective work environment. For example, Jones^36^ asks the question: “How is racism operating here”? Critical introspection, in this vein, means that anti-racism is not about wondering if racism is operating in a research setting, but asking “How is racism operating in this specific research setting?” and then embodying a willingness to actively listen to the answers and promote change.

### An anti-racism approach willingly sits with discomfort

How can there be a reckoning (deliberate truth-telling and resolute action) with racism without a willingness to sit with discomfort? Without discomfort, racism cannot be challenged. Anti-racism means recognizing that privilege, in particular white privilege, is a reality that has enabled some groups greater access to research opportunities and research training experiences that are not solely based on individual merit.^47^ We recognize this realization may be off-putting for some members of privileged groups.^48^ Yet, an anti-racism approach permits us to name it, confront it, own it, and embrace diversity as an ethos. It may be discomforting to know that there are scientists within minoritized communities who are as qualified as their white counterparts but whose individual merits are suppressed and denied.^49^ An anti-racism approach to research, in this sense, is also recognizing that many underrepresented scientists have been successful not solely because of diversity and institutional efforts but also because of their own self-determination and resilience in spite of structural barriers.

Overall, we argue that an anti-racism approach willingly grapples with these key considerations: (1) being intentional, (2) being critically introspective, and (3) sitting with discomfort. Without considering these components, diversity and equity are reduced, and, thus, equity in the scientific community will suffer and innovation may stagnate.^46^

## Moving forward

To truly advance diversity as an ethos, scientists in the field of genetics and genomics must start with introspection. Moving forward thus means examining the history of the field and where we are today. We do not provide “how-to” guidelines in this commentary. Instead, our recommendation is to start by intensifying the need for continuous reflection. To do this, we must value diversity beyond the numbers as well as recognize that diversity is not a favor. If we were to start from this point, then we would all ongoingly cultivate diversity as an ethos by willingly engaging with the reckoning of structural racism. This also means actively listening to the experiences of the many scientists (across the nation) who are directly and indirectly affected by structural racism. This is the intent of an anti-racism approach.

**Figure 1:**
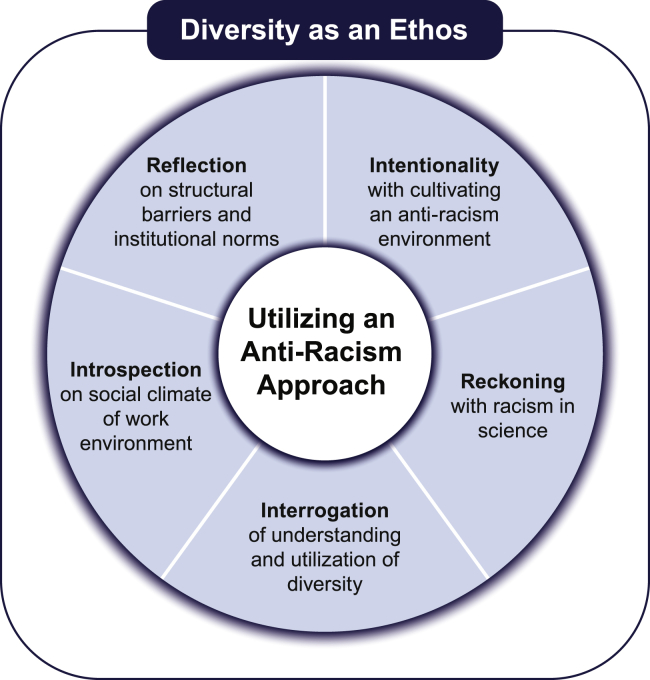
Conceptual model for diversity as an ethos utilizing an anti-racism approach

However, how do we begin to facilitate diversity as an ethos utilizing an anti-racism approach (Figure 1) in genomic science? First, we must recognize the conscious and unconscious practices^50^ of structural racism. We want to be clear: addressing structural racism does not mean that we should stop measuring and collecting data that evaluate racial differences in education, employment, housing, economics of community, and health outcomes.^51^^,^^52^ Measuring the use of race is indeed important to dismantling racial inequity. However, regardless of the ongoing study of race and racism, minoritized scientists are still experiencing the long-lasting effects of structural racism. This must be acknowledged if it is to be changed, because racism is real and has real consequences, as we are not in a post-racial America.^53^ Without critical introspection across the scientific research process, the same data used to substantiate the existence of racial inequality can also be used to subjugate people, perpetuate racism, and undermine the lived experience of racism.^54^ Therefore, we must continue collecting data that measure racism (and developing interventions to address structural racism). Furthermore, we must continue to reflect on the current social climate within the scientific workforce and the ongoing change needed in the field.

The goal of this commentary is to offer an opportunity to look at ourselves in the mirror and determine if we have done all we can to make the field more representative of the rich fabric of diversity of the US population.^55^ Each member of a research team is in a position to help foster diversity as an ethos. This will require individuals and research teams to engage in introspection and assess if they are building an equitable research environment that is oriented toward anti-racism. We call upon the field to have critical conversations (Box 1) to reflect on the questions. Reflecting on the questions (and the answers) will demonstrate how introspection functions as an inward process that can potentially guide efforts toward enhancing diversity as an ethos in the scientific enterprise.

Box 1 **Facilitating critical conversations within the scientific enterprise**

**Structural interrogation**

1. Studies show that certain groups are underrepresented in the research workforce. What barriers exist that contribute to this issue? What are some interventions to reduce these barriers?
2. How is genetics and genomics research used and misused to support or reinforce structural racism? How can genetics and genomics research be utilized to challenge structural racism?
3. What are the exclusionary and inclusionary norms in the scientific community? How are these norms maintained, ignored, or challenged within your research team?

**Personal reflection**

1. In your research team, is there a shared-understanding of racism? Discrimination? Prejudice? Anti-racism? How are these concepts and lived/personal experiences articulated and discussed by all members of the team?
2. Why do the members of your research team think that there are certain groups that are underrepresented in the research workforce?
3. If you are building a research team, what do you consider important when looking for potential members? How might the weight given to these factors be connected to biases or structural racism?
4. How is race used or not used in your studies, and why? How does your research team discuss race as a population descriptor, variable, or social category?

Box 1 provides a list of questions to facilitate critical conversation. Part one focuses on interrogation of structural systems, and part two provides the opportunity for personal reflection. We hope that these questions can serve as a tool to begin an important dialogue.

The questions in Box 1 are a starting point for the reader. We encourage you (and your research team) to come up with more questions, individually and collectively. The key point is about being willing to sit with the discomfort, while recognizing that the topic (and experience) of race and racism is discomforting for everyone involved. Discomfort, however, is also a core element of personal and professional growth. To achieve new possibilities for the field of genetics and genomics we all must cultivate diversity as an ethos, and we believe it requires an anti-racism approach.
